# Pest Protection Conferred by a *Beta vulgaris* Serine Proteinase Inhibitor Gene

**DOI:** 10.1371/journal.pone.0057303

**Published:** 2013-02-26

**Authors:** Ann C. Smigocki, Snezana Ivic-Haymes, Haiyan Li, Jelena Savić

**Affiliations:** United States Department of Agriculture, Agricultural Research Service, Molecular Plant Pathology Laboratory, Beltsville, Maryland, United States of America; Natural Resources Canada, Canada

## Abstract

Proteinase inhibitors provide a means of engineering plant resistance to insect pests. A *Beta vulgaris* serine proteinase inhibitor gene (*BvSTI*) was fused to the constitutive CaMV35S promoter for over-expression in *Nicotiana benthamiana* plants to study its effect on lepidopteran insect pests. Independently derived *BvSTI* transgenic tobacco T2 homozygous progeny were shown to have relatively high *BvSTI* gene transcript levels. BvSTI-specific polyclonal antibodies cross-reacted with the expected 30 kDA recombinant BvSTI protein on Western blots. In gel trypsin inhibitor activity assays revealed a major clear zone that corresponded to the BvSTI proteinase inhibitor that was not detected in the untransformed control plants. *BvSTI*-transgenic plants were bioassayed for resistance to five lepidopteran insect pests. *Spodoptera frugiperda*, *S. exigua* and *Manduca sexta* larvae fed *BvSTI* leaves had significant reductions in larval weights as compared to larvae fed on untransformed leaves. In contrast, larval weights increased relative to the controls when *Heliothis virescens* and *Agrotis ipsilon* larvae were fed on *BvSTI* leaves. As the larvae entered the pupal stage, pupal sizes reflected the overall larval weights. Some developmental abnormalities of the pupae and emerging moths were noted. These findings suggest that the sugar beet *BvSTI* gene may prove useful for effective control of several different lepidopteran insect pests in genetically modified tobacco and other plants. The sugar beet serine proteinase inhibitor may be more effective for insect control because sugar beet is cropped in restricted geographical areas thus limiting the exposure of the insects to sugar beet proteinase inhibitors and build up of non-sensitive midgut proteases.

## Introduction

Assimilation of dietary proteins is critical to normal insect growth and development, therefore, inhibition of digestive proteolytic enzymes is considered a desirable target for development of effective strategies to control insect pests. Insect digestive proteases are grouped into several mechanistic classes based on the amino acid residue or metal ion that is involved in peptide bond catalysis. Major midgut proteases of the Lepidoptera and Diptera insect orders been shown to be predominately of the serine (trypsin) type [1]–[3]. In the Homoptera and Coleoptera orders, major proteases utilized for digestion were shown to be of the cysteine class [4], [5]. These proteases are targeted by many naturally occurring plant proteinase inhibitors (PIs) that are similarly characterized by their specificity toward proteases [6]–[9]. PIs are considered attractive tools for crop improvement because their significant protective role in natural defense mechanisms has been well-documented [10]–[12]. Defensive capacities of plant PIs rely on inhibition of the insect’s digestive proteases thus limiting the availability of amino acids necessary for normal insect growth and development [4], [13].

Transfer of PI genes to plants is a widely accepted technique for engineering enhanced levels of insect tolerance in plants. It has been conclusively demonstrated that over-expression of heterologous PI genes significantly reduced or inhibited larval growth and feeding on transgenic plants [14]–[26]. The inhibition has been shown to be quite effective as demonstrated with bitter gourd PIs where more than 80% of *Helicoverpa armigera* serine proteases were inhibited by feeding on the transgenic PI plants [26]. Expression of rice cysteine PI genes, *oryzacystatin I* and *II*, was shown to increase resistance to several coleopteran pests, as well as nematodes, that commonly use cysteine proteases for protein digestion [23]–[25], [27]–[30]. In addition to insects, sweet potato and taro PI genes were shown to control microbial pathogens in tobacco [31]. In a reciprocal experiment where PI gene expression was suppressed in transgenic potato, an increase in larval weights of Colorado potato beetle (*Leptinotarsa decemlineata)* and beet armyworm (*Spodoptera exigua*) was reported [32].

One of the major challenges of the PI based insect control strategy has been the management of the inherent and induced complexity of the insect gut proteases. Since non-targeted proteases often can compensate for the blocked proteases, several approaches may be needed to combat this problem. One relatively recent approach was shown to be effective when tobacco and potato inhibitors of the same class were expressed simultaneously in the transgenic plant [33]. On the other hand, expression in tomato of two different classes of potato PI genes was shown effective for control of both a lepidopteran and a dipteran insect [14]. The potential to control more than one pest by gene stacking and for targeting nematodes and microbial pathogens makes the PI approach highly desirable for crop improvement. Clearly, however, the continued success of the PI based application strategy is dependent on the availability of newly discovered and characterized PI genes. PIs such as those derived from non-host plants to which the insect has had minimal or no prior exposure may prove most useful for enhancing insect resistance in engineered plants.

Extensive transcriptome and microarray studies are yielding a propensity of new knowledge about the classes of genes whose expression is modulated by plant-pest interactions. PI genes have often been found among the gene classes coupled to the defense response [34]–[37]. In our study of the interaction of the sugar beet root maggot (Tetanops myopaeformis Röder; Diptera:Ulidiidae) with the sugar beet root, a gene that encodes a serine PI (*BvSTI*) was found to be up-regulated in a sugar beet line with moderate resistance to the maggot [35], [38]. Since serine proteases comprise the major digestive enzymes in the root maggot midguts [39], these findings suggested that the *BvSTI* gene may be part of the overall resistance mechanism that protects the plant from insect attack. To investigate the potential function of the *BvSTI* PI gene in insect resistance, the gene was reconstructed for over-expression in transgenic plants. We report on the expression of the sugar beet *BvSTI* transgene in *N. benthamiana* plants and bioassay of the transgenic plants for insect resistance to five Lepidoptera insect pests.

## Materials and Methods

### Plant Transformation Vectors Carrying the *BvSTI* Gene

**Figure 1:**
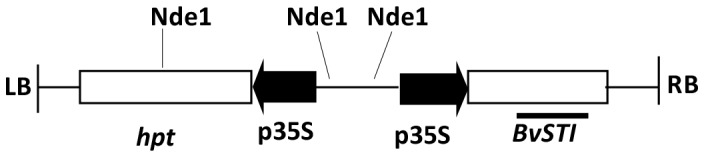
Schematic of the reconstructed *BvSTI* gene in the pCAMBIA1301 transformation vector (pBvSTI). RB, right border; LB, left border; p35S, cauliflower mosaic virus (CaMV) 35S promoter; *hpt*, hygromycin phosphotransferase selectable marker gene; NdeI restriction enzyme sites; arrows indicate direction of transcription from the p35S promoter. Horizontal bar indicates the 400-bp fragment of the *BvSTI* gene used as a probe for Southern blots.

The full length coding sequence of the *BvSTI* gene was obtained from the cloned EST sequence (GenBank #DV501688) using 5′ and 3′ RACE (BD Biosciences, San Jose, CA) and the following primers: 5′ RACE, 5′-CCATTTCTCAGTGCATCGCCGTCTGTGTCT-3′; and 3′ RACE, 5′-AGACACAGACGGCGATGCACTGAGAAATGG-3′ [40]. The full-length *BvSTI* gene was then amplified from sugar beet line F1016 [41] by RT-PCR using primers: Forward 5′ACCATGGCTTCCATTTTCCTGAAATC 3′ and Reverse 5′GGTCACCTAGACCATCGCTAAAACATCA 3′ that had an NcoI and BstEII restriction enzyme sites, respectively, built in for ease of sub-cloning. The full length *BvSTI* coding sequence was cloned behind the CaMV35S promoter in the pCAMBIA1301 plant transformation vector (pBvSTI, Fig. 1; CAMBIA, Canberra, Australia). pCAMBIA1301 carries the *hpt* marker gene for selection of hygromycin (Hg) resistant transformed plant cells.

### Plant Transformation

*N. benthamiana* (line 16c) leaf disks were excised and inoculated with *Agrobacterium tumefaciens* strain EHA105 that carries the pBvSTI transformation vector [38], [42]. Putative transformants were selected on Murashige and Skoog media containing B5 vitamins [43] and 20 mg Hg sulfate/l [38], [42]. Regenerated shoots were excised and placed on the same media for rooting prior to transfer to soil. After acclimation, plants were grown in the greenhouse and maintained at 20 to 30°C during the day and 18 to 25°C at night with a day length of 14 to 16 h. All plants were fertilized monthly with Osmocote (Scott’s Miracle-Gro, Marysville, OH). T2 progeny homozygous for Hg resistance were selected from the T1 progeny of independently derived T0 transgenic plants.

### Southern Blot Analysis

Genomic DNA was purified using the CTAB (hexadecyltrimethylammonium bromide, Sigma, USA) extraction method [44]. DNA concentration and purity were determined using an ND-8000 Spectrophotometer (NanoDrop Technologies Inc., DE, USA). Approximately 10 µg of DNA was digested with NdeI or NcoI restriction enzymes (New England Biolabs) that do not cut within the *BvSTI* gene construct, fragments were separated by electrophoresis on 1% agarose gels (Sigma, USA) and transferred to a positively charged nylon membrane (Roche, USA) in 10× SSC (8.76% NaCl and 4.41% sodium citrate, pH 7.0). Membranes were hybridized in DIG Easy Hyb (DIG High Prime DNA Labeling and Detection Starter Kit II, Roche) with DIG-labeled probes prepared using the PCR DIG Probe Synthesis Kit (Roche). To detect *BvSTI*, a 0.4 Kb of partial coding region fragment was used as probe. Detection of DIG probes was carried out as directed using CSPD Ready-to-Use (DIG-High Prime DNA Labeling and Detection Starter Kit II; Roche) and visualized on Lumi-film chemiluminescent detection film (Roche).

### RT-PCR

Total RNA was isolated using RNeasy Plant Mini Kit (Qiagen, USA) from approximately 100 mg of fresh leaf tissue and treated with RNase-free DNase (Qiagen, USA). Titanium One-Step RT-PCR Kit (Clontech Laboratories Inc., CA, USA) was used to amplify the *BvSTI* transgene transcripts from about 100 ng of total RNA under the following conditions: 50°C for 1 h, 94°C for 2 min 40 sec, 30 cycles of 94°C for 30 sec, 60°C for 40 sec, 72°C for 1 min 30 sec, followed by 72°C for 5 min. *BvSTI* gene specific primers described above were used to amplify the 0.6 Kb coding region [38]. To normalize RT-PCR results, transcripts of the constitutively expressed plant actin gene were used as loading controls. The following actin primers were used (Forward 5′-GTATTGTKAGCAACTGGGATGA-3′ and Reverse 5′-AACKYTCAGCCCRATGGTAAT-3′) to amplify a 0.54 Kb fragment using the same conditions as described above. RT-PCR analyses were repeated two times with comparable results.

### Protein Extraction

Native proteins were extracted in ice cold 50 mM Tris-HCl pH 7.5, 150 mM NaCl, 10 mM EDTA, 10% sucrose, 10 mM ascorbic acid, 1 mM PMSF, 2 mM DTT [38], [45]–[47]. Tissues were ground in liquid nitrogen and cold extraction buffer was added in proportion of 10 ml per 1 g of tissue. After centrifugation at 10000 rpm for 10 minutes, the supernatant (crude extract) was concentrated to about 1 ml using Amicon Ultra 15 (3K) concentrator (Millipore, USA) by centrifugation at 4°C. The concentrated extract was desalted in 8.5 ml of 62.5 mM Tris-HCl, pH 6.8 two times and centrifuged until the retentate volume was less than 200 µl. Total proteins were quantified according to Bradford (1976) [48].

### Western Blots

Total soluble proteins (15 or 30 µg) were separated on 12% SDS-PAGE gels in 0.025 M Tris, 0.192 M glycine and 3.5 mM SDS running buffer. After electrophoresis, gels were equilibrated in cold transfer buffer (0.025 M Tris, 0.192 M glycine, 0.025% SDS) for 1 hour. Separated proteins were subsequently transferred to Immun-Blot PVDF Membranes (0.2 µm, BioRad) for 1 hour 20 minutes at 70 V (Bio-Rad Mini-Trans-Blot Electrophoretic Transfer cell). Following transfer, membranes were rinsed in deionized water and gently agitated in blocking solution (5% BLOT-QuickBlocker, Chemicon International) for 1 hour. Membranes were then incubated with rabbit anti-BvSTI antibodies produced to a mixture of two most antigenic BvSTI peptides spanning amino acids 28–41 and 69–82 (GenScript Corporation, NJ, USA) [49] at 1∶2000 or 1∶5000 (v/v) dilutions in 1 x TBS-T (0.137 M NaCl, 0.02 M Tris pH 7.6, 0.1% Tween 20). After 1 hour 30 minutes incubation, membranes were rinsed two times in 1 × TBS-T for 10 minutes each, and incubated for 1 hour in alkaline phosphatase conjugated secondary antibody (AP Conjugated Goat anti-Rabbit IgG, 1∶5000 diluted in 1 × TBS-T, Chemicon International). Membranes were washed in 1 × TBS-T two times for 15 minutes and then 1 minute in 1 × TBS to remove the Tween 20. Alkaline phosphatase was detected using BCIP/NBT (5-bromo-4-chloro-3-indolylphosphate p-toluidine salt and nitro-blue tetrazolium chloride, respectively, Roche) in 0.1 M Tris–HCl pH 9.5, 0.1 M NaCl, and 0.05 M MgCl_2_ [42], [49]. Experiments were repeated two times.

### In-gel Trypsin Inhibitor Activity Assay

Native proteins (15 µg) were separated by SDS-polyacrylamide gel electrophoresis (SDS-PAGE). Gels were incubated with gentle shaking in 25% (v/v) 2-propanol, 10 mM Tris–HCl pH 7.4 for 30 minutes to remove SDS followed by 10 mM Tris–HCl pH 8.0 for another 30 minutes to renature the proteins [24], [38], [42], [46], [47], [50]. Gels were then soaked with 40 µg/ml bovine trypsin (Sigma) in 50 mM Tris–HCl pH 8.0, 50 mM CaCl_2_ for 40 minutes and transferred to a freshly prepared substrate-dye solution consisting of 2.5 mg/ml N-acetyl-DL-phenylalanine ß-naphthyl ester (Sigma) resuspended in dimethylformamide and 0.5 mg/ml tetrazotized O-dianisidine (Sigma) resuspended in 50 mM Tris–HCl pH 8.0 with 50 mM CaCl_2_, for 30 minutes at room temperature. Acetic acid (10%) was added to stop the reaction. Clear zones corresponding to proteins with trypsin inhibitory activity were recorded. Analysis was repeated three times with comparable results.

### Insect Feeding Bioassays

Newly emerged fall armyworm (*Spodoptera frugiperda* J.E. Smith), beet armyworm (*Spodoptera exigua* Hubner), black cutworms (*Agrotis ipsilon* Hufnagel) and tobacco budworm (*Heliothis virescens* Fabricius) larvae were purchased from Benzon Research (Carlisle, PA) and reared on the artificial diet provided by the supplier. Insects were maintained at room temperature for 1 to 3 days and removed from the diet 2 hours prior to start of experiments. For leaf assays, fully expanded leaves spanning approximately the middle third of 4-month old T2 homozygous greenhouse grown tobacco plants were used. Up to two leaves were removed from a plant. A single leaf was placed on water moistened filter paper in a Petri dish and infested with a weighed larva (second instar). Fresh leaves were added as needed. Water was added to the filters to prevent the leaves from drying out and wilting during the course of the experiment. Plates were kept in the dark at room temperature and larval weights and mortality were recorded daily until pupation. Experiments were repeated 2 to 5 times in reps of 5 to 10.

For whole plant experiments, a single transgenic *N. benthamiana* plant was placed in a screened cage and infested with a single third instar tobacco hornworm (*Manduca sexta* Linnaeus). Larvae were kindly provided by Lynda Liska (Agricultural Research Service, Beltsville, MD). Larval weights were recorded daily until pupation. Experiments were carried out in replicates of 3 to 5 plants and experiments were repeated 5 times.

Statistical analysis was performed by one-way Analysis of Variance (ANOVA) using Analyse-it software (Analyse-it Software, Ltd., Leeds, United Kingdom). Results are expressed as mean ± standard error (SE) for the number of replicates in each treatment. The acceptance level of statistical significance was *P*<0.05.

## Results

### *BvSTI* Gene Expression in Transgenic *N. benthamiana*

**Figure 2:**
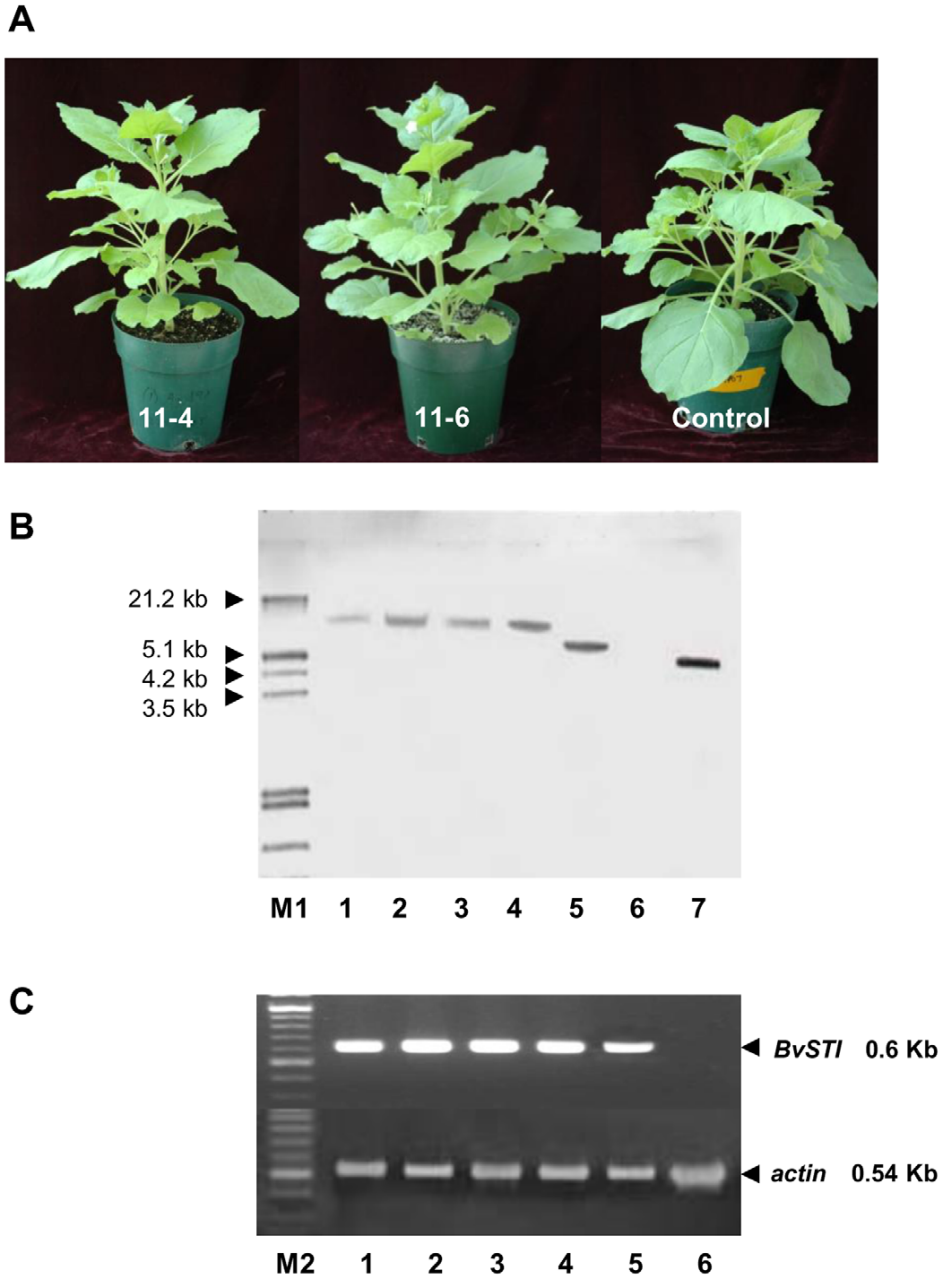
*N. benthamiana* plants transformed with the *BvSTI* gene. A) *N. benthamiana* 11-4 and 11-6 plants transformed with the *BvSTI* gene and normal untransformed plant**.** B) Southern blot of *N. benthamiana* plants. Lane 1–5, *BvSTI* transformant 11-4, 11-5, 11-6, 11-13 and 12-2, respectively; lane 6, untransformed normal control plant, lane 7, *BvSTI* plasmid DNA. All DNAs were digested with the NdeI restriction enzyme. M1, DNA molecular weight markers in kilobasepairs (Kb). C) RT-PCR analysis of transformants 11-4, 11-5, 11-6, 11-13 and 12-2 (lane 1–5, respectively) and normal untransformed control (lane 6) with *BvSTI* gene specific primers that amplify a 0.6 Kb gene fragment. *Actin*, housekeeping actin gene amplification with gene specific primers that generate a 0.54 Kb gene fragment (lane 1–6) used as quality and quantity control of the RNA being analyzed. M2, 100-bp DNA molecular weight markers.

The *BvSTI* gene codes for a sugar beet serine proteinase inhibitor that functions in the hydrolytic deactivation of trypsin proteases [38]. To analyze the function of the BvSTI PI in insect resistance, *BvSTI* was reconstructed for over-expression in transgenic *N. benthamiana* plants (Fig. 1). The independently derived T2 homozygous progeny exhibited phenotypes that were indistinguishable from the normal, untransformed control plants (Fig. 2A). Southern blot analysis of the T2 homozygous lines 11-4, 11-5, 11-6, 11-13 and 12-2 confirmed that a single copy of the *BvSTI* gene was integrated into the tobacco genome (Fig. 2B, lane 1–5, respectively). RT-PCR analysis revealed high levels of *BvSTI* gene transcripts in the transformants (Fig. 2C, lane 1–5 ) as compared to no detectable transcripts in the untransformed control (Fig. 2C, lane 6). Levels of *BvSTI* gene transcription were normalized to the constitutively expressed plant actin gene (Fig. 2C, lane 1–6).

### Western Blot Detection of BvSTI Protein

**Figure 3:**
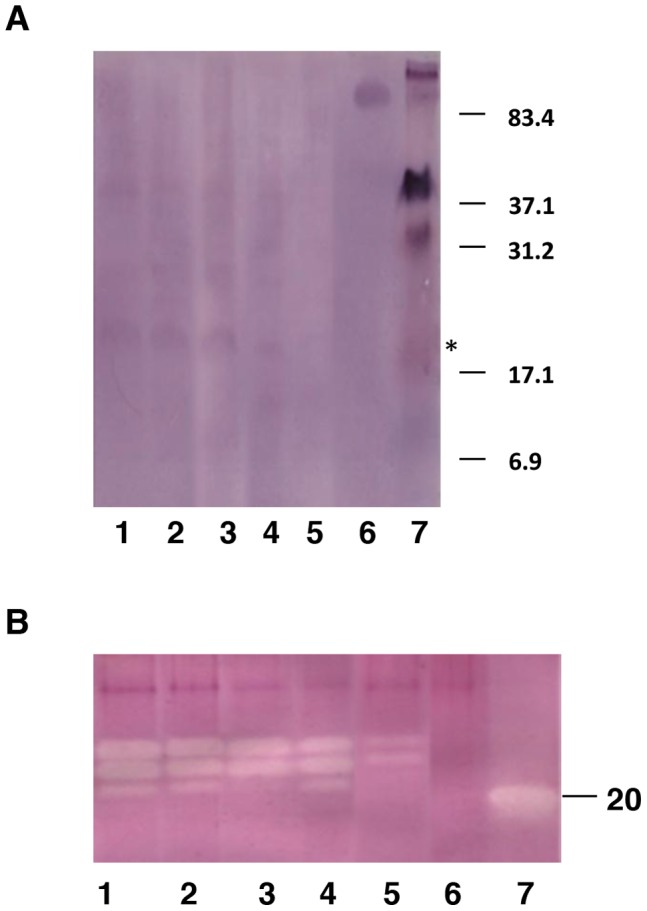
Immunoblot and in gel analysis of trypsin inhibitor activity in *N. benthamiana* plants transformed with the *BvSTI* gene. A) Immunoblot analysis of *BvSTI* transformed plants 11-4, 11-6, 11-13 and 12-2 (lane 1–4, respectively) and normal untransformed plant (lane 5) using BvSTI specific antibody. Lane 6, positive control for BvSTI peptides (5 ug of each peptide) used for production of the anti BvSTI-specific antibody; peptides were loaded 60 min after beginning of electrophoresis. Lane, 7, molecular weight standards in kDa. B). In gel analysis of trypsin inhibitor activity. Lane 1–5, *BvSTI* transformant 11-4, 11-5, 11-6, 11-13 and 12-2, respectively; lane 6, untransformed normal control plant; lane 7, positive control, soybean Kunitz trypsin inhibitor protein, 20 kDa, 0.5 µg.

Presence of the recombinant protein in the T2 transformed lines was confirmed by Western blot analysis with BvSTI specific polyclonal antibodies (Fig. 3A) [49]. Weak protein signal of about 30 kDa as well as a stronger signal in the range of 22–25 kDa cross-reacted with the BvSTI antibodies in the transgenic 11-4, 11-6, 11-13 and 12-2 tobacco plants (Fig. 3A, lane 1–4). Overall, protein concentrations that cross-reacted with the BvSTI specific antibody were low in all of the analyzed transformants and no obviously cross reacting proteins in the 22–30 kDa range were detected in transformant 11-5 (data not shown) or the untransformed control (Fig. 3A, lane 5).

### BvSTI Proteinase Inhibitor Activity

To determine the level of BvSTI proteinase inhibitor activity, total protein extracts from transgenic leaves were analyzed by an in-gel trypsin inhibitor activity assay. Multiple clear zones (white bands) corresponding to trypsin inhibitor activity of approximately 30, 28 and 26 kDa were detected in T2 transformants 11-4, 11-5, 11-6, 11-13 and 12-2 (Fig. 3B, lane 1–5, respectively) that were not observed in the untransformed control plant (Fig. 3B, lane 6). In addition to the 30 kDa BvSTI protein activity previously observed in sugar beet, two additional zones of activity corresponding to approximately 28 and 26 kDa were clearly visible in line 11-4, 11-5 and 11-13 (Fig. 3B, lane 1, 2 and 4) [49]. Transformant 11-6 had reduced level of the active 26 kDa trypsin inhibitor (Fig. 3B, lane 3) compared to 11-4, 11-5 and 11-13. Line 12-2 had the lowest levels of the active 30 and 28 kDa proteins and no detectable 26 kDa activity (Fig. 3B, lane 5).

### Bioassays on *Spodoptera Frugiperda* Larvae

**Figure 4:**
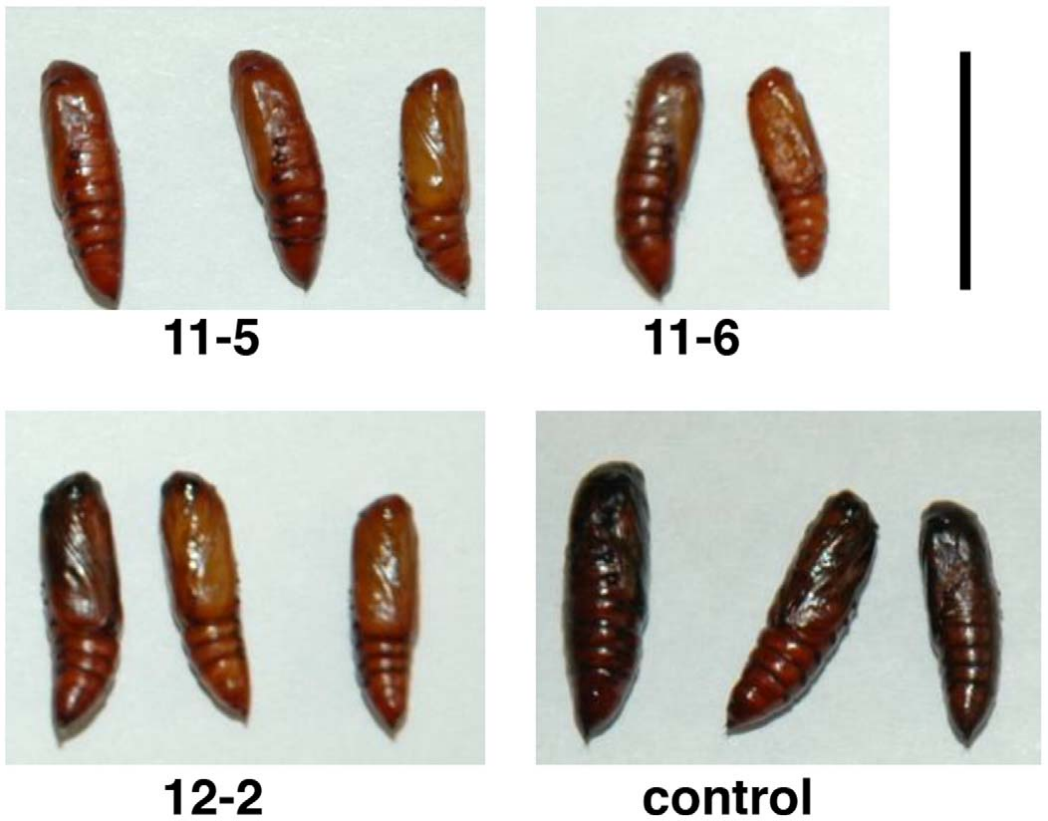
Fall armyworm pupae from feeding bioassays on *N. benthamiana* plants transformed with the *BvSTI* gene. Transformant 11-5, 11-6 and 12-2 and untransformed normal control plant. Bar corresponds to 1 cm.

Insect feeding assays were conducted to study the effect of the sugar beet BvSTI proteinase inhibitor on growth and development of several Lepidoptera insects. The five independently derived *N. benthamiana* transgenic plants that showed high levels of *BvSTI* gene expression and detectable hydrolytic trypsin activity were bioassayed for resistance to fall armyworm, a generalist lepidopteran herbivore with a wide host range. Second-instars were fed detached transgenic or non-transgenic leaves and daily observations were made to determine survival, weight gain and developmental stage of the larvae. Larvae were weighed at the start of the experiment and only ones with non-significantly different weights were used in the bioassay. Larvae feeding on leaves from *BvSTI* transformed plants 11-4, 11-5, 11-6, 11-13 and 12-2 had significantly reduced mean larval weights at 3 (31 to 43 mg; except line 12-2), 6 (48 to 95 mg) and 8 (74 to 105 mg; except line 12-2) days as compared to the control larval weights of 63, 143 and 258 mg, respectively (Table 1). At 10 days, larval weights of the controls were reduced because some larvae started to pupate, unlike the larvae feeding on the transformants. In general, a 1 to 3 day delay in onset of pupation was observed for larvae feeding on the *BvSTI* transformed leaves. Pupal sizes reflected the overall larval weights at pupation, i.e. smaller for the ones feeding on transgenic leaves, and lighter brown in color as compared to the larger and darker controls (Fig. 4). The rate of insect emergence from pupal case for the treatment or the control seemed comparable and all moths had a similar appearance. Experiments were repeated two more times and significantly reduced larval weights were observed at day 3, 5, 6, 7 and 8 for larvae feeding on the *BvSTI* transformants (data not shown). No significant differences in larval mortality rates were noted (Table 1). At day 3, 6 and 8, larval mortality averaged 4, 16 and 25% for the transformants as compared to 0, 13 and 27% for the controls, respectively.

**Table 1:** Weights of fall armyworm larvae feeding on *N. benthamiana* T2 homozygous plants transformed with the *BvSTI* gene.

Transformants	3 days	6 days	8 days	10 days
11-4	31±4.1^a^ (15)	48±9.2^a^ (11)	76±15.9^a^ (9)	131±33.6^a^ (8)
11-5	43±6.5^a^ (15)	70±8.2^a^ (14)	105±13.0^a^ (13)	162±23.1^b^ (12)
11-6	32±3.1^a^ (13)	52±6.4^a^ (11)	74±9.9^a^ (11)	106±16.5^a^ (10)
11-13	39±3.2^a^ (14)	55±8.4^a^ (13)	84±9.3^a^ (11)	112±12.0^a^ (9)
12-2	51±7.8^b^ (15)	95±19.2^a^ (14)	157±35.5^b^ (12)	183±38.0^b^ (11)
Control	63±7.7^b^ (15)	143±23.9^b^ (13)	258±42.2^b^ (11)	234±25.4^b^ (8)

Values represent mean larval weight ± SE (mg).

Means followed by the same superscript within columns are not significantly different (*P*<0.05) by one-way ANOVA test. Number in parenthesis indicates the number of living larvae out of 15 that were weighed.

### Bioassay on *Spodoptera exigua* Larvae

**Figure 5:**
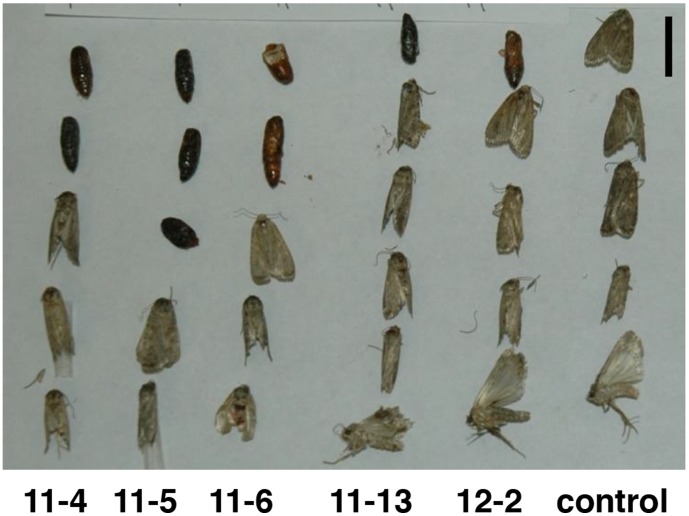
Beet armyworm pupae and emerged moths from feeding bioassays on *N. benthamiana* plants transformed with the *BvSTI* gene. Transformant 11-4, 11-5, 11-6, 11-13 and 12-2 and untransformed normal control plant. Bar corresponds to 1 cm.

Similar studies were done with beet armyworm to examine the effect of the *BvSTI* proteinase inhibitor gene on larval growth and development. Larval weights were reduced at 5 and 7 days of feeding on *BvSTI* transformed plants 11-4, 11-5, 11-6, 11-13 and 12-2 when compared to larval weights on the control. However, the reduced weights were only significant on larvae feeding on *BvSTI* transformant 11-4 and 11-5 at 5 days (87 and 88 mg compared to 139 mg for the control; Table 2). In a repeat experiment, all larval weights were similarly reduced, however, only the larvae feeding on transformants 11-6 and 11-13 had significant reduction in their weights (179 and 190, respectively compared to 233 for the control; data not shown). No significant differences in larval mortality or pupation were noted. A higher incidence of pupae displaying some abnormal development and non-emergence was observed when larvae were fed transgenic leaves (Fig. 5).

**Table 2:** Weights of beet armyworm larvae feeding on *N. benthamiana* T2 homozygous plants transformed with the *BvSTI* gene.

Transformants	0 days	5 days	7 days
11-4	38±2.0^b^ (8)	87±13^a^ (7)	116±27^b^ (6)
11-5	38±2.0^b^ (8)	88±15^a^ (7)	108±18^b^ (6)
11-6	36±2.0^b^ (8)	109±18^b^ (8)	183±30^b^ (7)
11-13	38±2.0^b^ (8)	109±9.2^b^ (8)	160±25^b^ (5)
12-2	36±1.0^b^ (8)	108±13^b^ (8)	125±23^b^ (7)
Control	37±1.0^b^ (8)	139±20^b^ (8)	168±27^b^ (7)

Values represent mean larval weight ± SE (mg).

Means followed by the same superscript within columns are not significantly different (*P*<0.05) by one-way ANOVA test. Number in parenthesis indicates the number of living larvae out of 8 that were weighed.

### Bioassay on *Manduca sexta* Larvae

**Figure 6:**
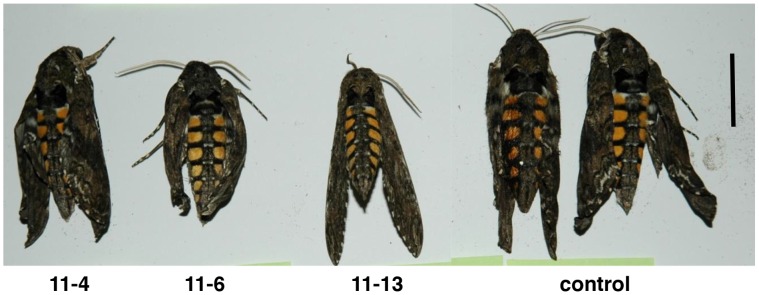
Tobacco hornworm moths after feeding on *N. benthamiana* plants transformed with the *BvSTI* gene. Transformant 11-4, 11-6 and 11-13 and untransformed normal control plant. Bar corresponds to 2 cm.

A whole plant bioassay of the *N. benthamiana* transgenic plants was conducted with third instar tobacco hornworm. At 4, 6 and 10 days after start of the bioassay, all larvae feeding on the *BvSTI* transformants 11-4, 11-6 and 11-13 had significant lower weights than the larvae fed control except for transformant 11-6 at day 4 and 10 (Table 3). At day 6, average larval weights ranged from 1.5 to 1.9 g for the transformants as compared to 3.7 g for the controls. In repeat experiments, weights of larvae feeding on transformant 11-6 were significantly reduced (3.1 g) as compared to the control (5.1 g) at 7 days. No differences in larval mortality were noted and pupal sizes reflected larval weights. Some abnormal wing development and smaller body sizes were noted on the emerged moths fed on the BvSTI transformants (Fig. 6).

**Table 3:** Weights of tobacco hornworm larvae feeding on *N. benthamiana* T2 homozygous plants transformed with the *BvSTI* gene.

Transformants	0 days	4 days	6 days	10 days
11-4	0.3±.02^b^ (5)	1.0±0.1^a^ (5)	1.9±0.3^a^ (5)	5.0±0.8^a^ (5)
11-6	0.3±.01^b^ (5)	1.1±0.2^b^ (5)	1.9±0.3^a^ (5)	6.1±0.9^b^ (5)
11-13	0.3±.01^b^ (5)	0.8±0.1^a^ (5)	1.5±0.2^a^ (5)	4.5±1.0^a^ (5)
Control	0.3±.01^b^ (5)	1.5±0.2^b^ (4)	3.7±0.5^b^ (4)	8.1±0.6^b^ (4)

Values represent mean larval weight ± SE (g).

Means followed by the same superscript within columns are not significantly different (*P*<0.05) by one-way ANOVA test. Number in parenthesis indicates the number of living larvae out of 5 that were weighed.

### Bioassay on *Agrotis ipsilon* Larvae

**Figure 7:**
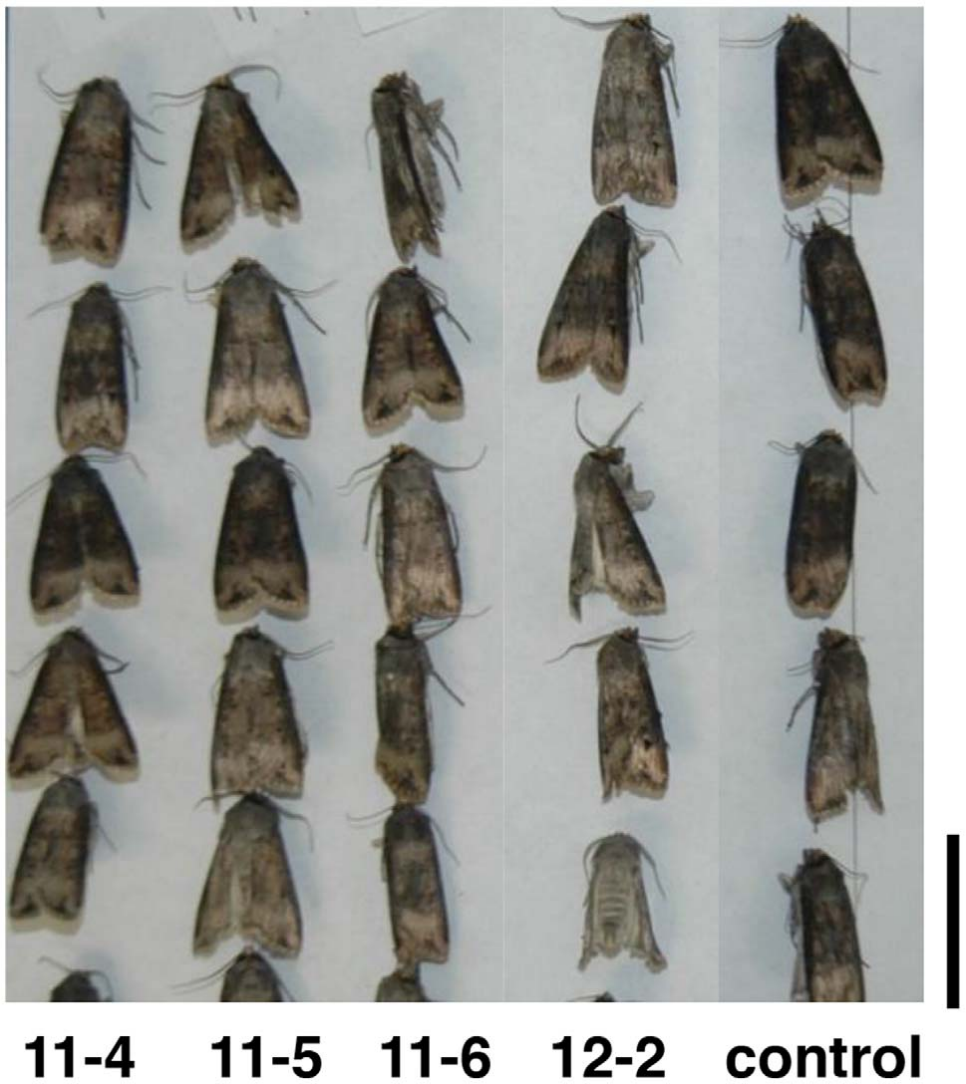
Black cutworm moths after feeding on *N. benthamiana* plants transformed with the *BvSTI* gene. Transformant 11-4, 11-5, 11-6 and 12-2 and untransformed normal control plant. Bar corresponds to 2 cm.

Black cutworm larvae were fed leaves from 11-4, 11-5, 11-6, 11-13 and 12-2 transgenic *BvSTI* plants. At 3, 5 and 7 days, average larval weights on all five *BvSTI* transformants were higher than those of the larvae that fed on the control leaves (Table 4). Average larval weights at 3 days ranged from 116 to 158 mg and were significantly higher than the control larval weights of 63 mg, except for larvae feeding on *BvSTI* transformant 11-6 (116 mg). At 5 days, larval weights ranged from 141 to 202 mg for the treatments and 81 mg for the control with larval weights after feeding on 12-2 being significantly higher. Similar increases in larval weights were also noted at 7 days, averaging around 282 mg for the treatment as compared to 197 for the control. In repeat experiments, similar increases in larval weights were noted for the transgenic treatments as compared to control. No differences in larval mortality were noted among the larvae feeding on the transgenic leaves (Table 4) and pupal sizes reflected the increased larval weights, as did the emerging moths (Fig. 7).

**Table 4:** Weights of black cutworm feeding on leaves from *N. benthamiana* BvSTI T2 homozygous plants.

Transformants	3 days	5 days	7 days
11-4	136±22^a^ (5)	173±37^b^ (5)	330±87^a^ (5)
11-5	129±24^a^ (5)	165±37^b^ (5)	266±75^a^ (5)
11-6	116±8.4^b^ (5)	168±18^b^ (5)	299±30^a^ (5)
11-13	128±20^a^ (5)	141±31^b^ (4)	202±59^a^ (4)
12-2	158±31^a^ (5)	202±18^a^ (4)	315±36^a^ (4)
Control	63±36^b^ (4)	81±39^b^ (3)	197±0 (1)†

Values represent mean larval weight ± SE (mg).

Means followed by the same superscript within columns are not significantly different (*P*<0.05) by one-way ANOVA test. Number in parenthesis indicates the number of living larvae out of 5 that were weighed.

† data was not included in the statistical analysis at 7 days since only 1 larvae was weighed, the other 4 pupated.

### Bioassay on *Heliothis Virescens* Larvae

**Figure 8:**
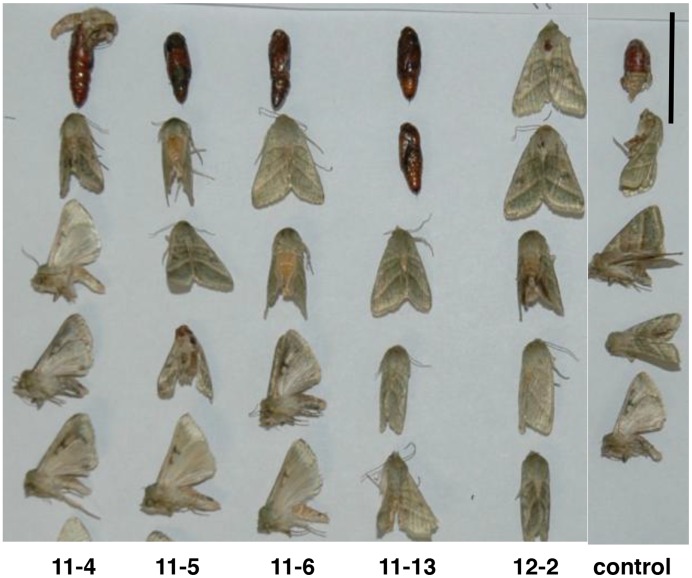
Tobacco budworm larvae and emerged moths from feeding bioassays on *N. benthamiana* plants transformed with the *BvSTI* gene. Transformant 11-4, 11-5, 11-6, 11-13 and 12-2 and untransformed normal control plant. Bar corresponds to 2 cm.

Tobacco budworm larvae were fed leaves from *BvSTI* transgenic plants 11-4, 11-5, 11-6, 11-13 and 12-2. At 5 and 7 days after the start of the feeding assay, all larvae feeding on the BvSTI transformants were heavier than the larvae feeding on the controls (Table 5). At 5 days, larval weights ranged from 172 to 237 mg (average 200 mg per larvae) for the treatments as compared to 159 mg for the control. At 7 days, larval weights ranged from 221 to 276 mg (average 235 mg) for the larvae fed transformed leaves as compared to 191 mg for the control. Increases in larval weights were significant for larvae fed on transformant 12-2. In two separate repeat experiments, similar increases in larval weights were observed for the transgenic treatments as compared to the control. Some differences in larval mortality rates were noted. Larvae fed on 11-5 and 11-6 transformants had mortality rates of 3 out of 10 and 2 out of 10, respectively, and for 11-13 and 12-2 the rate was 5 out of 10 as compared to 1 out of 10 for the control. Some varying degrees of developmental abnormalities of the wings and aborted insect emergence were observed (Fig. 8).

**Table 5:** Weights of tobacco budworm feeding on leaves from *N. benthamiana* BvSTI T2 homozygous plants.

Transformants	5 days	7 days	12 days †
11-4	172±14^b^ (10)	221±16^b^	183±21^b^ (9)
11-5	196±20^b^ (10)	239±18^b^	390±162^b^ (7)
11-6	198±13^b^ (10)	217±15^b^	206±23^b^ (8)
11-13	199±20^b^ (10)	221±21^b^	180±29^b^ (5)
12-2	237±17^a^ (10)	276±15^a^	209±6^b^ (5)
Control	159±15^b^ (10)	191±16^b^	198±16^b^ (9)

Values represent mean larval weight ± SE (mg).

Means followed by the same superscript within columns are not significantly different (*P*<0.05) by one-way ANOVA test.

† Larvae started to pupate at 9 days; Number in parenthesis indicates the number of living larvae out of 10 at start of experiment.

## Discussion

Plants have an assortment of defensive genes whose products harm insects and pathogens. Among the mechanisms of plant defense are genes that in response to wounding lead to the expression of proteinase inhibitors that disrupt protein digestion in insect midguts. Over-expression of heterologous PI genes in crop plants has resulted in enhanced resistance to a wide spectrum of insect pests. However, the ongoing challenge of the PI based insect control strategy is the need to discover and characterize new and novel PIs to address the inherent and induced complexity of the insect gut proteases. PIs such as those derived from non-host plants to which the targeted insect has had minimal or no prior exposure can generally be most useful for enhancing insect resistance in engineered plants. In a study of sugar beet root defense responses, a single serine PI gene (*BvSTI*) was identified among the more than 150 sugar beet genes whose expression was found to be modulated by a dipteran pest of sugar beet, the root maggot [35]. Expression of the *BvSTI* gene was determined to be up-regulated by mechanical- and insect-wounding in sugar beet lines used in breeding for root maggot resistance [41], [49]. The observed lack or reduced accumulation and activity of BvSTI PI in tissues of susceptible and less resistant lines emphasized the potentially important role of the BvSTI PI in insect pest defense mechanisms. In this study, the prospect of over-expressing the sugar beet *BvSTI* gene for control of lepidopteran insect pests in genetically modified *N. benthamiana* was investigated. Serine proteases that include trypsin-, chymotrypsin- and elastase-like have been well-documented as comprising the major midgut proteolytic activities in lepidopteran insects [3], [51], [52]. Homozygous T2 populations of transgenic *N. benthamiana* plants carrying a single copy of the *BvSTI* transgene construct exhibited phenotypes that were similar to the normal untransformed plants (Fig. 2A and B). Elevated levels of *BvSTI* gene transcripts driven by the constitutive CaMV35S promoter were detected in all analyzed T2 homozygous plants (Fig. 2C). Presence of the recombinant BvSTI proteins in the T2 transformants was confirmed on Western blots with BvSTI-specific antibody that cross-reacted with low quantities of peptides in the range of 22–25 kDa and 30 kDa that was previously observed in sugar beet (Fig. 3A) [49]. These finding suggests that processing and modification of the recombinant BvSTI protein may be different in the tobacco background as compared to its regulation in sugar beet. Detection of low levels of recombinant PI protein has been reported by others [21]. Independently derived apple transformants with increased resistance to the light-brown apple moth had low levels of the recombinant PI protein [21]. It has also been shown that feeding inhibition did not necessarily increase beyond that observed with low protein concentrations in studies where recombinant PI proteins were fed to larvae [53]. Because the detected signal on Western blots was weak this suggests a possible high turnover and/or modification of the BvSTI protein in *N. benthamiana* irrespective of the high transcript and trypsin protein activity levels in the BvSTI transformants (Fig. 2C and 3B). A unique and distinct clear zone of at about 30 kDa was detected in all five homozygous *BvSTI* transformants by an in gel trypsin activity assay (Fig. 3B). Two additional activity zones corresponding to proteins of approximately 28 and 26 kDa were also visible in the transformed lines but not in the untransformed control plants (Fig. 3B). No apparent cross-reactivity of the BvSTI specific antibody with the 28 and 26 kDa trypsin inhibitory proteins was observed on Western blots although one can argue that the 26 kDa protein is similar to that in the range of 21–25 kDa (Fig. 3A). All three of these active proteins may represent varying degrees of modification in the tobacco genetic background that are different than what was observed in the sugar beet background except for the 30 kDa protein [49]. The smaller proteins may represent modified or partially degraded forms of the 30 kDa BvSTI protein subjected to proteolytic enzymes of the host plant. A possibility that these proteins represent newly induced proteinase inhibitors of *N. benthamiana* cannot be excluded.

A number of the independently drived *BvSTI*-transgenic plants were bioassayed for resistance to several lepidopteran insects that are pests of tobacco. Fall armyworm, beet armyworm, tobacco hornworm, tobacco budworm and black cutworm cause significant yield losses in hundreds of economically important crops and all, with the exception of tobacco hornworm and budworm, infest sugar beet. The sugar beet root maggot was not included in this study since its host range is limited and does not include *Nicotiana* spp. The major digestive proteases utilized by the lepidopteran insects in this study have been reported to be predominantly in the serine class of proteases [3]. Therefore, presence of the recombinant BvSTI proteinase inhibitor has the potential to deter insect feeding or inhibit digestion of ingested food thus reducing the overall larval weights as compared to larvae feeding on untransformed control plants. When the *BvSTI*-transgenic plants were fed to fall armyworm, beet armyworm and tobacco hornworm larvae, significant reductions in larval weights were observed, with some pupae and emerging moths displaying developmental abnormalities. Fall armyworm larvae weighed 19–51%, 34–66% and 59–71% less at 3, 6 and 8 days of feeding, respectively, as compared to control larvae (Table 1). Except for the smaller pupae sizes that corresponded to the reduced larval weights and a lighter brown color, no significant differences in development or mortality rates were noted (Fig. 4). The beet armyworm pupae and emerging moth sizes similarly reflected the reduced weights of the larvae fed the *BvSTI* transgenic leaves. In addition, many of the pupae did not emerge as moths and of the ones that did, developmental abnormalities were often noted (Fig. 5). Tobacco hornworm larvae were also significantly smaller than the control larvae and the resulting pupae and moth sizes correlated with the reduced larval weights (Fig. 6). Larval weights after 6 days of feeding on the *BvSTI* transgenic plants were about 50–60% lower than those fed on control untransformed plants (Table 3).

In contrast, black cutworm and tobacco budworm larvae fed on *BvSTI* transformed plants accumulated biomass faster than those fed on the control foliage. Black cutworm larval weighs were more than double those of the control larvae at 3 and 5 days of feeding (Table 4). After 7 days, the larvae weighed almost 50% more than the control larvae. No differences in larval mortality were noted and pupae and moth sizes reflected larval weights (Fig. 7). Similar responses were observed with tobacco budworm larvae fed on *BvSTI* leaves. On the average, the larvae were 10 to 50% heavier than the control larvae (Table 5). Larval mortality rates were up to 5 times those of the control larvae and emerging moths displayed varying degrees of abnormal wing development after feeding on the BvSTI transformants (Fig. 8). Increases in larval weights feeding on proteinase inhibitor transformed plant materials have been reported by others [14], [54]–[56]. Faster biomass accumulation of Colorado potato beetle feeding on potato transformed with a rice cysteine proteinase inhibitor gene (*OCI*) was reported [54]–[56]. A similar increase in larval weights with potato transformed with another rice cystatin gene, *OCII,* was also noted (data not shown). This can be expected if the dynamics of protein hydrolysis are changed due to proteinase inhibition or modified proteinase profiles. Thus, a modified nutritional status could trigger persistent hunger in larvae and result in compensatory feeding. Also, in order to extract sufficient nutritional benefits needed to sustain growth and development, larval feeding (and weight) would have to increase to compensate for the inhibition. Others have also suggested that exposure to sub-lethal concentrations of proteinase inhibitors may have induced a similar compensatory response in *Heliothis obsolete* and *Liriomyza trifolii* that led to increased feeding and faster larval growth [14]. Similarly, the possibility that enhanced performance may not be related to the inhibitory action of the recombinant PIs was also proposed [54], [57].

Our findings suggest that the sugar beet *BvSTI* gene should prove useful for effective relatively broad spectrum control of lepidopteran insect pests. We demonstrated that production of the recombinant BvSTI PI in transgenic *N. benthamiana* reduced weights of feeding larvae of three of the five lepidopteran pests and increased larval weights of the other two that we tested. The expectation is that any variation in weight, be it a decrease or an increase, may alter the normal life cycle of the insect thus changing the dynamics and timing of the interaction with the host plant, a desirable strategy for enhancing insect tolerance. Since some developmental abnormalities of the pupae and the emerging moths were also noted, BvSTI treatment appeared to have a negative effect on the insect’s morphogenesis, a strategy for successful control. The basis for the increased resistance may be due to the limited exposure of these insect pests to sugar beet and, in this case specifically, the BvSTI serine proteinase inhibitor. Since sugar beet is generally grown in geographically limited areas, these insects are less likely to have developed digestive proteases resistant to the sugar beet serine proteinase inhibitor. Further studies are needed to define the effect of the BvSTI PI on other insects, including ones of different orders. Also, the functional role of the recombinant BvSTI PI in crops other than *N. benthamiana* is yet to be determined. Similarly, since it has been reported that the defensive function of PIs in *N. attenuata* sometimes relies on synergistic effects with other defenses such as nicotine and herbivory-induced plant volatiles (HIPVs), these factors have to be considered in the design of transgenic crop species that express PIs for field applications [58], [59]. Clearly the defensive effect of one or several transgenic PIs in a crop species will most likely depend on the metabolite background and the herbivore/predator community of the transformed host species. We propose that the *BvSTI* gene in combination with other PI or resistance transgenes may provide a useful strategy for improving resistance in economically important crop species that would benefit from improved insect tolerance.
